# Integrative hologenomic analysis reveals apolipoprotein D–associated host–microbe metabolic crosstalk linked to fat deposition in Jinhua pigs

**DOI:** 10.3389/fmicb.2026.1915318

**Published:** 2026-09-16

**Authors:** Ran Shen, Shuang Liu, Pengfei Yu, Shuyan Xie, Qinqin Xie, Qishan Wang, Yuchun Pan, Zhe Zhang, Zhen Wang, Wei Zhao

**Affiliations:** 1 Zhejiang Key Laboratory of Nutrition and Breeding for High-Quality Animal Products, College of Animal Sciences, Zhejiang University, Hangzhou, China; 2 Key Laboratory of Livestock and Poultry Resources Evaluation and Utilization, Ministry of Agriculture and Rural Affairs, Hangzhou, China; 3 Hainan Institute of Zhejiang University, Yongyou Industrial Park, Yazhou Bay Science and Technology City, Sanya, China; 4 Hainan Yazhou Bay Seed Lab, Yongyou Industrial Park, Yazhou Bay Sci-Tech City, Sanya, China; 5 SciGene Biotechnology Co., Ltd, Hefei, China

**Keywords:** fat deposition, gut microbiota, hologenomic, host–microbe interaction, Jinhua pig

## Abstract

**Background:**

Adipose deposition is a complex trait shaped by both host genetics and environmental factors, including the gut microbiota. While quantitative trait loci (QTLs) and candidate genes underlying fat accumulation have been extensively identified in diverse pig breeds, the hologenomic regulatory networks integrating host genetic variation and microbial signals remain to be fully deciphered.

**Methods:**

In this study, we utilized a strictly controlled dietary cross-fostering model involving Jinhua and Landrace×Yorkshire pigs across developmental stages (day60, day90 and day180). We integrated population genetics, eQTL analysis, transcriptomics, metagenomics and metabolomics to elucidate the host-metabolite-microbe axis.

**Results:**

Phenotypic analysis revealed a robust genetic trait for high backfat thickness in Jinhua pigs, independent of dietary intervention. Through *F*_ST_ analysis and eQTL mapping, we pinpointed a strongly differentiated locus (13_133481393_A_G) that was nearly fixed in JH pigs (allele frequency 0.95) and was associated with upregulation of *APOD*. In our multi-omics dataset, this locus was linked to shifts in cecal gene expression that were consistent with a pro-adipogenic program, which coincided with an intestinal metabolic profile marked by elevated oleic acid and reduced oleoylethanolamide. This metabolic niche, in turn, directionally recruited synergistic microbes, specifically *Eubacterium* sp. *AM28-29* and *Blautia*, which functioned as biochemical amplifiers to further accelerate lipid accumulation.

**Conclusion:**

Our study establishes a hologenomic framework demonstrating how foundational genetic selection (*APOD*) shapes the host-microbe metabolic interplay to drive complex production traits. This study offers novel molecular targets and a theoretical framework for precision breeding and nutritional modulation of meat quality in livestock.

## Graphical abstract

**Figure:**
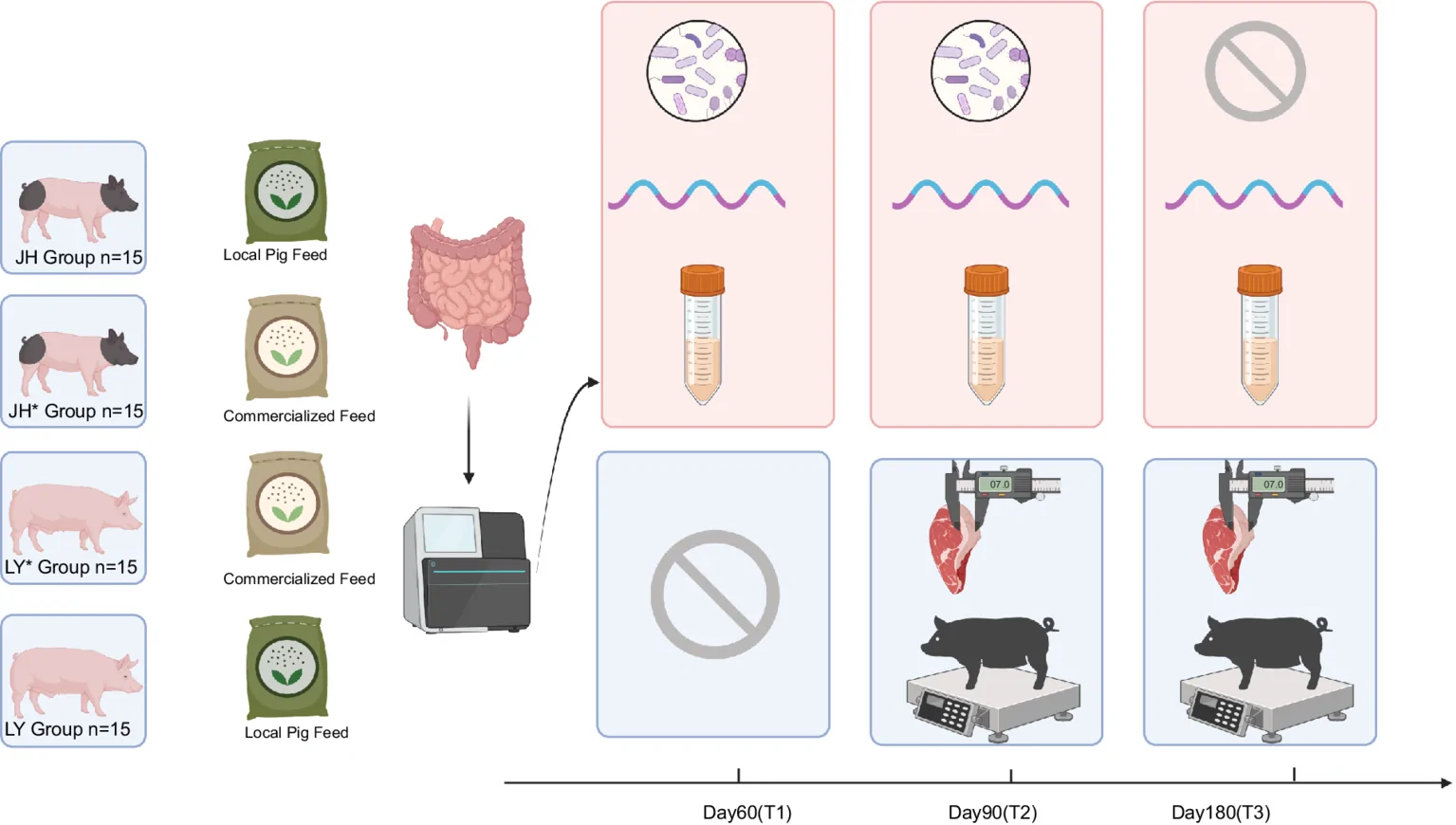

## Introduction

Driven by evolving consumer preferences, the focus of modern pig production has shifted from mere quantity and growth efficiency to the enhancement of product quality (Scollan et al., 2017). European commercial breeds, represented by Landrace and Yorkshire, have undergone intensive artificial selection aimed at rapid weight gain (Xing et al., 2019). However, this has concomitantly led to a decline in meat quality attributes such as tenderness, juiciness and flavor (Zhang et al., 2022). Specifically, the intramuscular fat (IMF) content in lean commercial pig breeds is typically around 1%, which falls below the 2.2–3.4% range required for optimal palatability (Font-i-Furnols et al., 2013; Fortin et al., 2005; Zhao et al., 2025). In contrast, the Jinhua (JH) pig, a highly acclaimed Chinese indigenous breed renowned for producing the world-class Jinhua ham, exhibits exceptional IMF and marbling performance (Xiao et al., 2018). In recent years, comparative studies between Chinese indigenous fat-type pigs and commercial lean-type pigs have preliminarily revealed the pivotal role of lipid metabolism in meat quality improvement (Xiao et al., 2018; Kumar et al., 2025; Yang et al., 2025). Nevertheless, the underlying genetic architecture and regulatory networks governing the superior meat quality traits of JH pigs remain to be fully elucidated.

With the rapid advancement of high-throughput technologies, multi-omics sequencing has emerged as a robust tool for investigating complex traits controlled by genetic and environmental factors, such as meat quality (Zhang W.-T. et al., 2025). In this context, the role of the gut microbiota in modulating host fat deposition is garnering increasing attention. Evidence suggests that specific gut microbial taxa can influence host lipid metabolism via their bioactive metabolites (Lu and Stappenbeck, 2022). For instance, riboflavin-synthesizing bacteria such as *Lachnospiraceae* activate lipid remodeling in mesenteric adipocytes by secreting flavin adenine dinucleotide (FAD), whereas *Phascolarctobacterium succinatutens* promotes arginine synthesis via propionate production, thereby inhibiting fat deposition (Tong et al., 2026; Lan et al., 2025). The regulatory roles of the gut microbiota are often deeply intertwined with the host’s genetic background, and studies have confirmed the existence of complex cross-kingdom interactions between the two. For instance, in yellow broilers, fat deposition is co-regulated by the host and the cecal microbiota, including *Methanobrevibacter* and *Mucispirillum schaedleri* (Wen et al., 2019). In murine models, colonization by *Prevotella copri* activates chronic inflammatory responses via the *TLR4* and mTOR signaling pathways, significantly upregulating the expression of genes associated with adipogenesis and fat accumulation while concurrently suppressing those related to lipolysis, lipid transport, and muscle growth (Chen et al., 2021). These cross-species evidence indicate that fat deposition is mutually driven by the interplay between host genetics and the microbiome. To systematically capture these intricate interactions, the holobiont concept has emerged, framing the host and its associated microorganisms as a unified biological entity to decipher the genetic mechanisms underlying complex traits (Kobel et al., 2024).

Within the porcine gut microbiome, the cecum serves as a core organ for microbial aggregation and fermentation, making it a critical target for studying host–microbe interactions (Han et al., 2021). The cecum harbors a dense and diverse microbial community capable of facilitating the fermentation of non-digestible polysaccharides, yielding bioactive metabolites such as short-chain fatty acids (SCFAs) and secondary bile acids (Yu et al., 2024; He et al., 2023). Serving as systemic signaling molecules, these metabolites can bind to host G-protein-coupled receptors to modulate energy homeostasis and adipogenesis (Koh et al., 2016). Consequently, characterizing the composition and function of the cecal microbiome, alongside its interactions with host genes, is of great significance for uncovering the mechanisms driving the adipogenic advantages of JH pigs. However, from a hologenomic perspective, systematic multi-omics evidence regarding how the JH pig cecal microbiome and host genome synergistically interact to shape its unique fat deposition characteristics remains scarce. Although the continuous decline in sequencing costs has facilitated the accumulation of massive public datasets, which provides a valuable multi-omics resource derived from large pig populations—mining these diverse data sources presents significant challenges. Specifically, it is difficult to effectively control for confounding variables such as rearing environment, dietary formulation, and sampling age. Consequently, the multi-omics data generated under standardized environmental and dietary conditions are essential to minimize biological noise and uncover the fundamental drivers of physiological variation.

Therefore, in the present study, we employed Jinhua (JH) and Landrace × Yorkshire (LY) pigs reared under standardized environmental conditions with controlled reciprocal dietary treatments as a comparative model to dissect the hologenomic basis of divergent fat deposition. We hypothesized that the robust adiposity phenotype of JH pigs is governed by a host-initiated hologenomic axis, in which breed-specific genomic selection drives coordinated differences in host gene regulation, intestinal metabolite composition, and gut microbial community structure, collectively establishing a pro-adipogenic microenvironment. To test this hypothesis, the present study pursued four specific objectives: (1) to characterize the phenotypic stability of fat deposition traits in JH pigs across reciprocal dietary conditions; (2) to identify breed-specific gut microbial taxa and intestinal metabolites robustly associated with backfat thickness independent of dietary background; (3) to resolve host colonic transcriptomic co-expression networks correlated with key microbial taxa and to identify candidate genetic drivers under breed-specific genomic selection; and (4) to integrate population genomic, transcriptomic, metagenomic, and metabolomic data within a hologenomic framework to characterize the potential interrelationships among host genetics, gut microbiota, and intestinal metabolism underlying breed-divergent fat deposition. Collectively, this study aims to provide new mechanistic insights into host–microbiome associations with fat deposition and to establish a scientific basis for future precision breeding and targeted nutritional strategies to improve pork quality.

## Results

### The fat deposition trait of Jinhua pigs shows strong genetic robustness

To elucidate the genetic mechanisms and host-nutrition interactions governing divergent fat deposition in JH and LY pigs, we conducted longitudinal slaughter trials at 60 (T1), 90 (T2) and 180 (T3) days of age (Figure 1A). Under standardized environmental conditions, pigs were initially fed according to their respective breed-specific nutritional standards (*n* = 5 per breed/stage). To isolate the impact of dietary factors, a reciprocal diet-swap treatment was implemented at each stage using an additional five pigs per breed, during which cecal tissues, contents, and phenotypic data were harvested. Groups fed the LY diet were marked with an asterisk (*).

**Figure 1:**
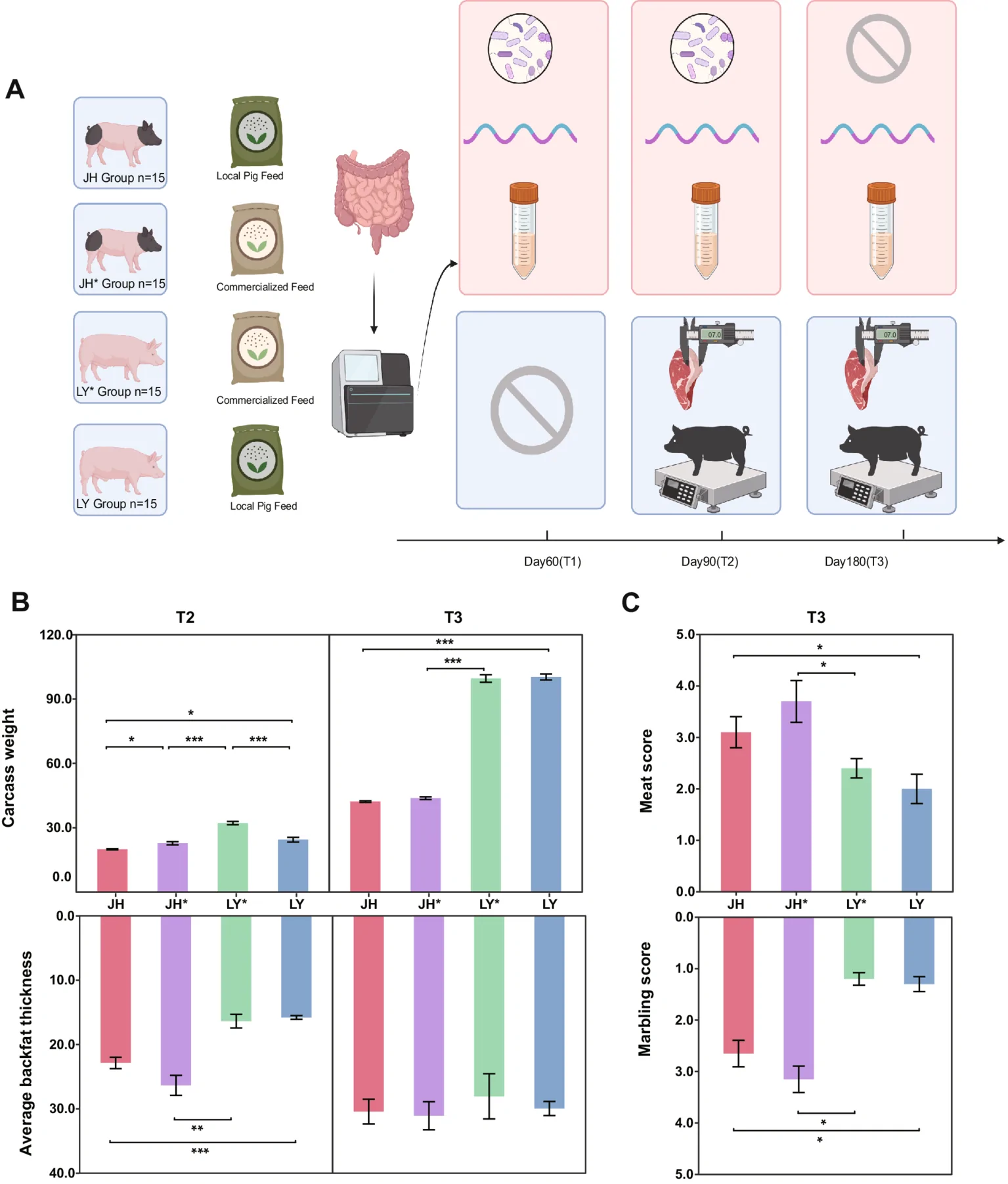
Phenotypic characterization of pigs from different genetic backgrounds and experimental design. **(A)** Experimental group design. **(B)** Carcass weight and average backfat thickness. **(C)** Meat color score and marbling score. *, ** and *** indicate *p* < 0.05, *p* < 0.01 and *p* < 0.001, respectively.

At the T2 stage, although the carcass weight of JH pigs was significantly lower than that of LY pigs, their backfat thickness (BFT) was significantly higher (*p* < 0.05) (Figure 1B; Supplementary Table 1). By the T3 stage, the carcass weight of LY pigs reached approximately 2.5 times that of JH pigs, yet BFT remained comparable between the two breeds, highlighting a pronounced phenotypic advantage for lipid deposition in JH pigs (Figure 1B). Three-way ANOVA demonstrated that BFT was primarily driven by breed and age, with no significant influence from dietary changes (F_breed = 15.9937, *p* = 0.00035; F_time = 55.052, *p* = 1.924e-08; F_diet = 0.297, *p* = 0.59) (Supplementary Table 1). In contrast, while carcass weight was co-regulated by breed, age, and diet, dietary interventions failed to narrow the breed-specific gap in carcass weight under our experimental conditions. Furthermore, the significant superiority of JH pigs in marbling scores underscores their exceptional lipid deposition potential, a trait that appears to be deeply regulated by breed factors (*p* < 0.05) (Figure 1C). Collectively, these phenotypic patterns suggest that fat deposition in JH pigs is under strong genetic control that is not readily disrupted by dietary perturbation.

### Breed-specific microbiota succession and selective regulation of lipid traits

The principal coordinates analysis (PCoA) based on Bray-Curtis distances indicated that community succession was alternately driven by genetic background and dietary components. During the T1 stage, the LY diet promoted a convergence in microbial community composition between breeds (*p* = 0.082) (Figure 2A). However, upon T2 stage, the host genetic effect reclaimed dominance over the microbiota architecture (*p* < 0.05) (Figure 2A). Consistent with the phenotypic observations, these results suggest that host genetics exert a dominant influence on the core microbial framework, maintaining its structural integrity despite dietary variations (Figure 2B). To identify key taxa driving these breed differences, we employed the multivariate association linear model (MaAsLin2). After filtering for low abundance (< 0.0001%) and low prevalence (< 50%), 62 breed-associated microbial features were identified (Figure 2C). Notably, 34 bacteria, including *Eubacterium* sp. *AM28-29*, *Blautia obeum, Blautia wexlerae*, *Holdemanella biformis* and *Floccifex porci* were significantly enriched in JH pigs, primarily belonging to genera such as *Eubacterium*, *Blautia*, and *Ruminococcus* (Supplementary Table 2). Correlation analysis further revealed that JH-enriched *Eubacterium* sp. *AM28-29, Blautia obeum*, *Ruminococcus* sp. *JC304* and *Blautia wexlerae* were significantly positively correlated with BFT but showed no significant association with carcass weight (Figure 2D). This suggests that these specific strains might mediate the unique lipid deposition traits of JH pigs through the precise regulation of fat deposition rather than by promoting overall growth.

**Figure 2:**
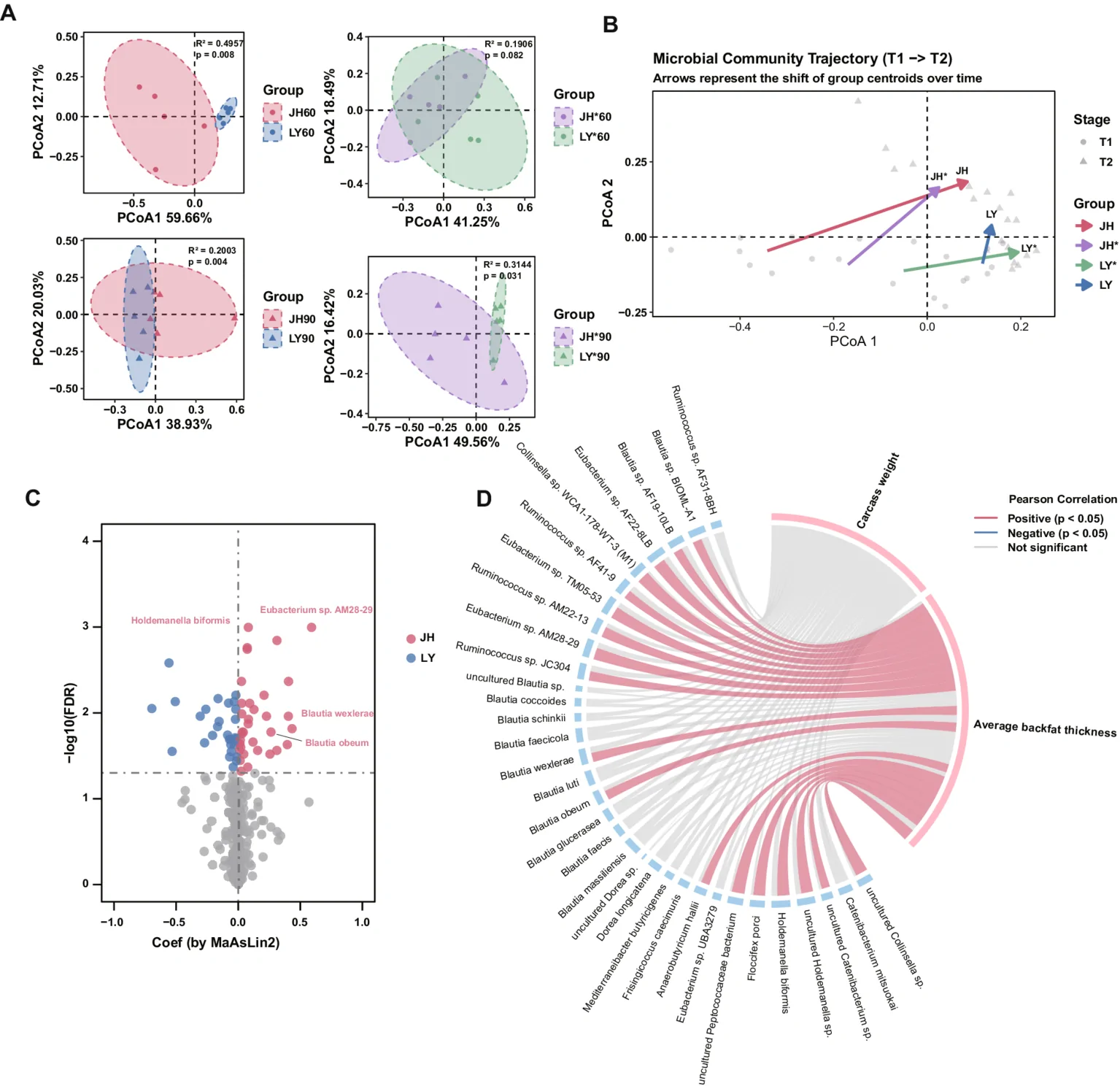
Breed and diet effects on gut microbiota composition, temporal dynamics, and associations with carcass traits in pigs. The PCoA based on Bray–Curtis dissimilarity showing **(A)** distinctive microbial community structure, and **(B)** group centroid trajectories from T1 to T2. Arrows indicate the direction and magnitude of microbial community shifts over time. **(C)** The coefficient values of MaAsLin2 model responding to breed factor in combined all time points dataset. **(D)** Pearson’s correlations coefficients of breed-specific microbiota with carcass weight and backfat thickness.

### Identification of robust metabolic signatures linked to fat accumulation

To isolate core metabolic features of fat deposition, we focused on the T2 stage, where phenotypic data was comprehensive, and screened for differential metabolites under both dietary interventions (VIP > 1, *p* < 0.05) (Figure 3A). Out of 1,627 annotated metabolites, 194 and 145 differential metabolites were identified under the JH and LY diets, respectively (Supplementary Table 3). An intersection strategy yielded 32 robust breed-specific metabolites (Figure 3B). After excluding glycochenodeoxycholic acid and taurochenodeoxycholic acid due to inconsistent expression patterns across diets, 30 core metabolites were retained. Association analysis revealed a divergence in the effects of these robust metabolites on growth versus adiposity, with 24 metabolites exhibited opposite correlation patterns (Figure 3D). For instance, L-(−)-malic acid was significantly positively correlated with BFT but negatively correlated with carcass weight. Other metabolites exhibited strong phenotypic specificity, correlating exclusively with BFT. Notably, the primary lipid synthesis substrate, oleic acid (OA), was consistently elevated in the intestines of JH pigs, whereas the lipid signaling molecule oleoyl ethanolamide (OEA), known for regulating energy expenditure, was significantly lower in JH pigs compared to LY pigs.

**Figure 3:**
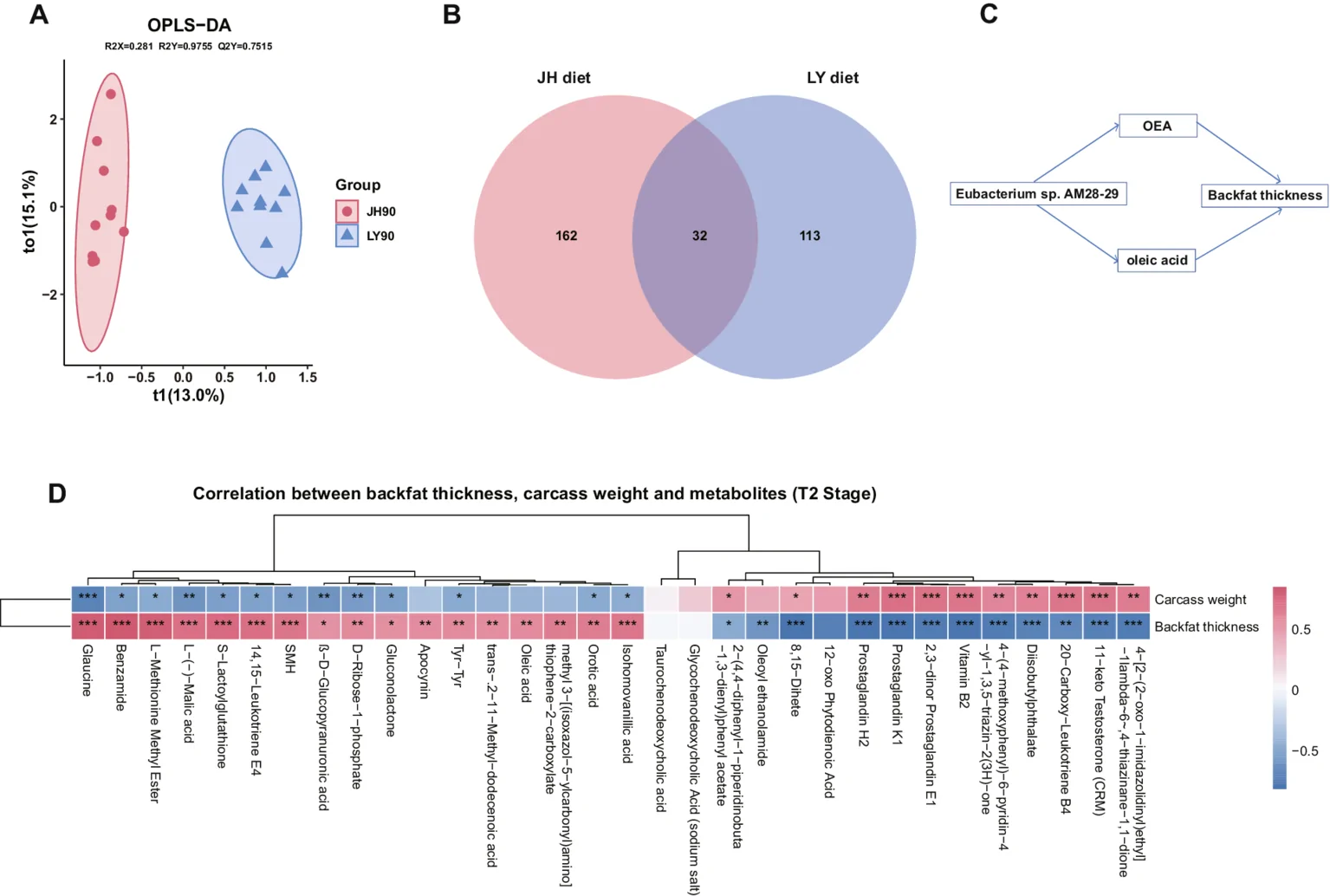
Multi-omics integration reveals breed-specific metabolic signatures and their mediation of carcass traits in pigs. **(A)** The OPLS-DA of metabolites between JH and LY pigs under positive electrospray ionization (ESI+) mode. **(B)** Venn diagram showing the intersection of differentially abundant metabolites between JH- and LY-diet groups at T2 time point. **(C)** Mediation analysis of breed-specific metabolites associated with backfat thickness and core metabolites. **(D)** Pearson correlation analysis between core metabolites and carcass weight and backfat thickness.

To decipher the mechanistic link between the core microbiota and fat deposition, we performed mediation analysis to test whether specific metabolites bridge the association between *Eubacterium* sp. *AM28-29* and BFT (Supplementary Table 4). The results revealed a dual-metabolite mediation model. On one hand, *Eubacterium* sp. *AM28-29* promoted lipid accumulation by increasing the availability of oleic acid (mediation effect: 29.7%). On the other hand, the fat-promoting effect of this bacterium may be mediated by reduced OEA levels (mediation effect: 24.6%) (Figure 3C). These results suggest that *Eubacterium* sp. *AM28-29* accelerates fat deposition through an indirect axis by simultaneously promoting lipid synthesis precursors and inhibiting catabolic signaling pathways.

### Co-expression network analysis reveals lipid metabolism modules associated with gut microbiota

To systematically dissect the host genetic regulatory networks underlying fat deposition, we first analyzed the colonic transcriptome across the T1, T2, and T3 developmental stages. Principal component analysis (PCA) revealed that breed identity remained the predominant driver of transcriptional variation throughout the developmental cycle, whereas dietary intervention had limited impact, underscoring the profound genetic robustness of JH pig adiposity traits (Figure 4A). Differential expression analysis identified a clear functional shift across development. JH-upregulated differentially expressed genes (DEGs, |log₂FC| ≥ 1, FDR < 0.05) were primarily involved in viral infection and immune responses during T1, while transitioning toward lipid metabolism pathways in T2 and T3 (Supplementary Table 5). To further resolve synchronized gene expression patterns and their cross-kingdom interactions with the gut microbiota, we performed WGCNA, identifying 13 functionally distinct co-expression modules. The green-yellow module (60 genes) emerged as a key regulatory unit (Supplementary Table 6). Module-trait association analysis showed significant positive correlations between this module and several JH-enriched microbes, most notably *Eubacterium* sp. *AM28-29* (r = 0.45, *p* = 4e-04) (Figure 4B). Functional enrichment localized this module to critical lipid synthesis and energy metabolism pathways, including PPAR signaling, fat digestion and absorption, and cholesterol metabolism (Figure 4C). Notably, the intersection of MEgreenyellow module member genes and the DEGs dentified ENSSSCG00000038322, apolipoprotein D (*APOD*), diacylglycerol O-acyltransferase 2 (*DGAT2*), and secreted frizzled-related protein 5 (*SFRP5*) as genes satisfying both co-expression and differential expression criteria. While the module membership only partially overlapped with the total DEGs set, their biological signatures exhibited remarkable functional convergence. Specifically, the fat digestion and absorption and cAMP signaling pathways were consistently enriched in both the stage-specific DEGs and the microbial-linked co-expression module (Figures 4D,E).

**Figure 4:**
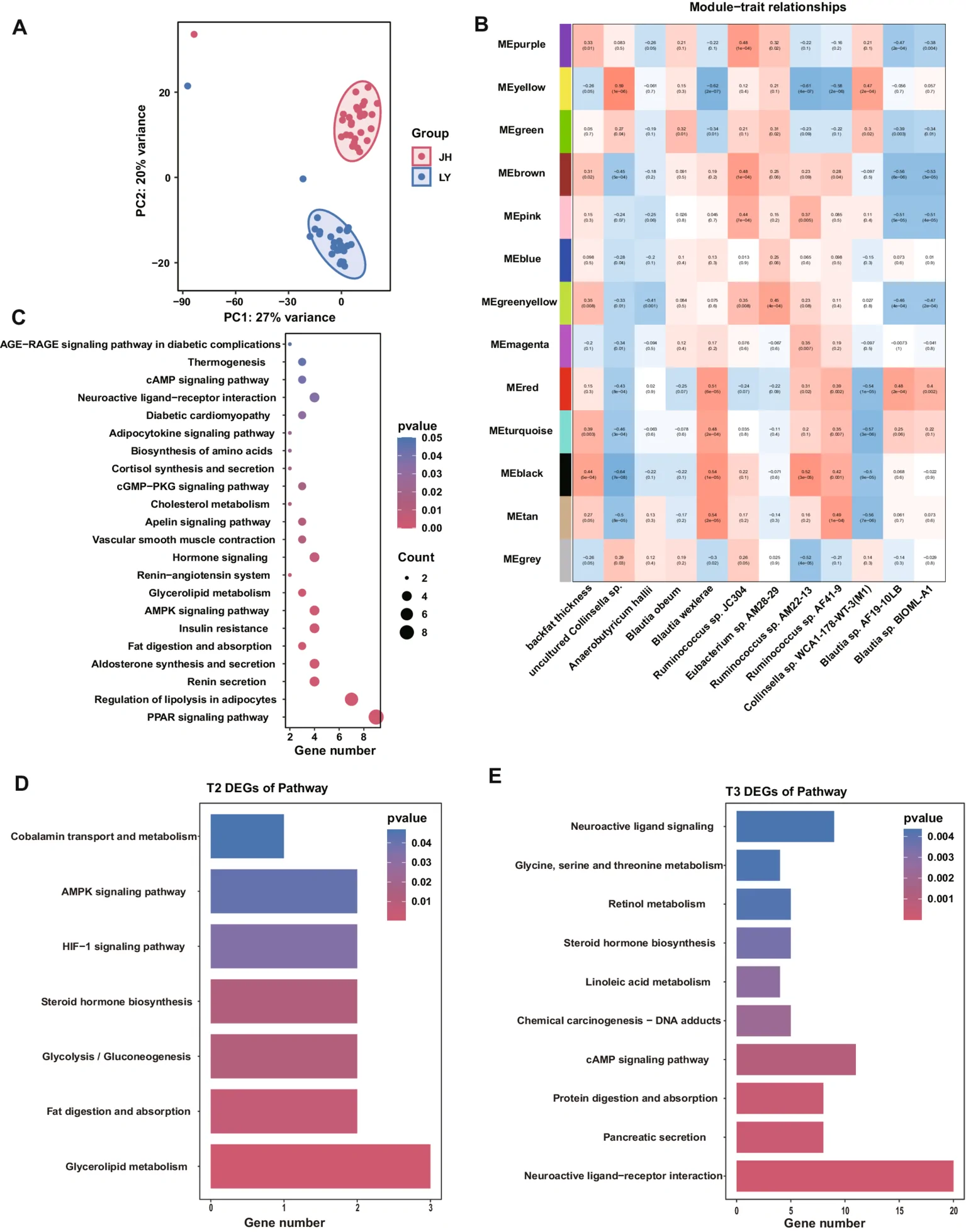
Integration of adipose transcriptome dynamics and gut microbiota associations across growth stages in pigs. **(A)** The PCA of subcutaneous adipose tissue transcriptomes at T1, T2 and T3 developmental stages. **(B)** Correlation matrix between weighted gene co-expression network analysis (WGCNA) modules and core gut microbial taxa. **(C)** KEGG pathway enrichment analysis of genes in the green yellow module (key microbiota-associated module). KEGG enrichment of differentially expressed genes (DEGs) identified at T2 **(D)** and T3 **(E)**.

Integration of adipose transcriptome dynamics and gut microbiota associations across growth stages in pigs. (A) The PCA of subcutaneous adipose tissue transcriptomes at T1, T2 and T3 developmental stages. (B) Correlation matrix between weighted gene co-expression network analysis (WGCNA) modules and core gut microbial taxa. (C) KEGG pathway enrichment analysis of genes in the green yellow module (key microbiota-associated module). KEGG enrichment of differentially expressed genes (DEGs) identified at T2 (D) and T3 (E).

### Host genome–specific selection drives divergence in the transcriptome

Given the pronounced transcriptomic divergence observed between JH and LY pigs, we hypothesized that the underlying driving force stems from breed-specific genomic selection. By intersecting the DEGs with population Fixation index (*F*_ST_), we identified five overlapping genes: *APOD*, *PPL*, *SDR16C5*, *GPX3* and *CA3*. Given its direct functional role in lipid metabolism, *APOD* was prioritized as the primary candidate gene (Figure 5A; Supplementary Table 7; Steyrer and Kostner, 1988). The eQTL analysis from the PigGTEx database (n = 1,324, muscle) confirmed that this SNP (13_133481393_A_G, effect size = 0.249097, *p* = 3.959797 × 10^−6^) positively modulated *APOD* transcript levels, acting as a potential regulatory element (Figure 5B; Supplementary Table 8). In the JH population, the allele frequency of G at this locus was 0.95, whereas the LY population was fixed for the homozygous AA genotype at the 13_133481393 site (Figure 5C). This genomic differentiation aligns with the divergent expression patterns and lipid-related phenotypes of the two breeds. To elucidate the complex interplay among host genetics, gut microbiota and the metabolic microenvironment, we performed an integrated multi-omics analysis using the DIABLO (Data Integration Analysis for Biomarker discovery using Latent variable approaches for Omics proteins) framework. The resulting circos plot and relevance network (Figures 5D,E) revealed a highly interconnected hologenomic network centered on the genetic anchor *APOD.* Specifically, *APOD* expression exhibited strong positive correlations with both *Eubacterium* sp. *AM28-29* abundance and OA levels. Conversely, OEA was negatively correlated with both *APOD* and *Eubacterium* sp. *AM28-29*. These findings suggest that the intestinal balance of OA and OEA is jointly orchestrated by the host genetic locus *APOD* and the key microbial taxon *Eubacterium* sp. *AM28-29*, potentially driving the divergent fat deposition phenotypes.

**Figure 5:**
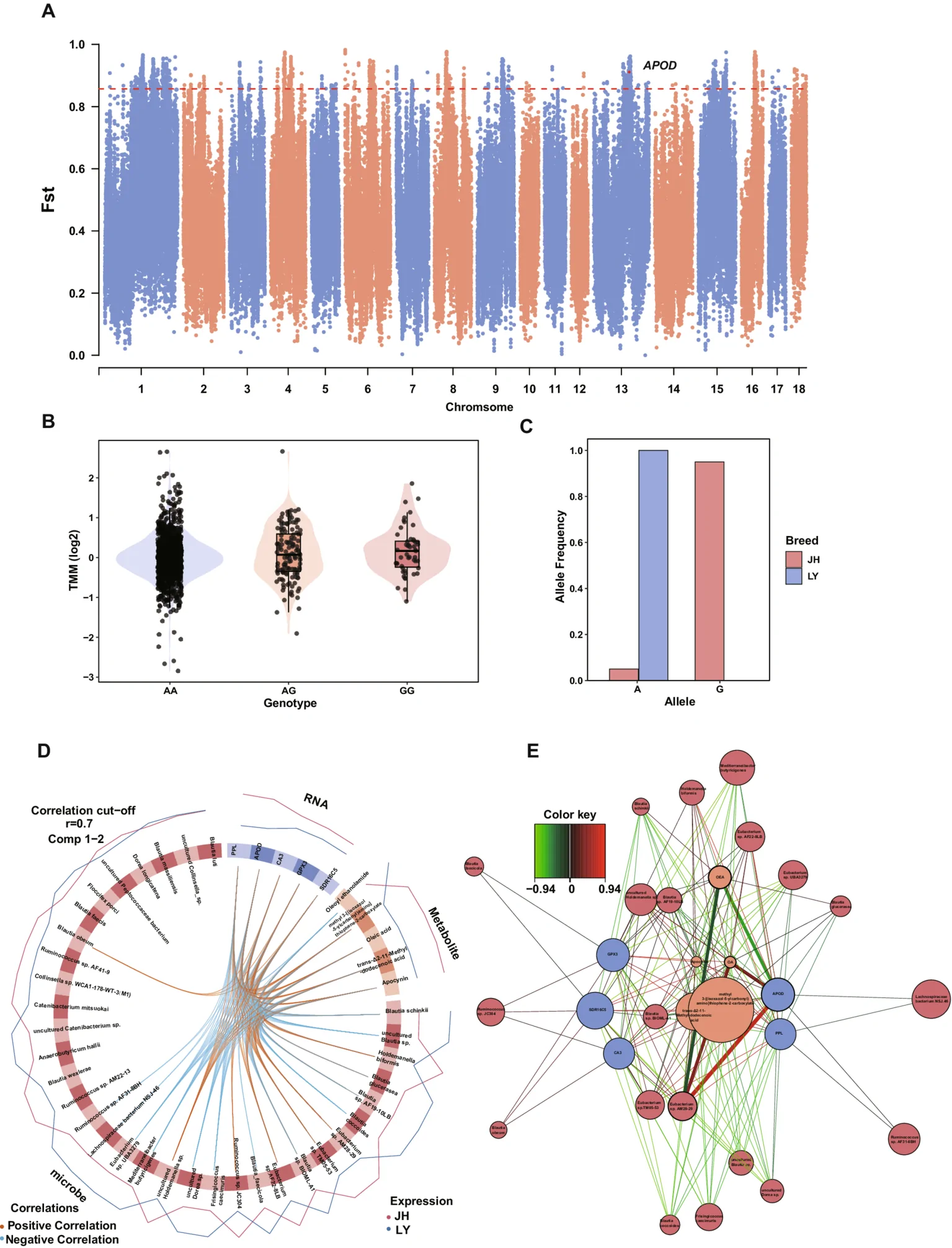
Identification validation of a breed-specific regulatory variant affecting *APOD* expression in pigs. **(A)** Manhattan plot showing *F*_ST_-based selective signals on chromosome 13, with the peak region harboring *APOD* highlighted. **(B)** The eQTL mapping demonstrating the regulatory effect of variant 13:133481393_A_G on *APOD* gene expression in muscle tissue (data from PigGTEx). **(C)** Allele frequency comparison of the 13:133481393_A_G variant between JH and LY pigs. **(D)** Circos plot highlighted correlation (*r* > 0.7) between variables from the different omics blocks. **(E)** Relevance network of the multi-omics signatures.

Identification validation of a breed-specific regulatory variant affecting *APOD* expression in pigs. (A) Manhattan plot showing *F*_ST_-based selective signals on chromosome 13, with the peak region harboring *APOD* highlighted. (B) The eQTL mapping demonstrating the regulatory effect of variant 13:133481393_A_G on *APOD* gene expression in muscle tissue (data from PigGTEx). (C) Allele frequency comparison of the 13:133481393_A_G variant between JH and LY pigs. (D) Circos plot highlighted correlation (r > 0.7) between variables from the different omics blocks. (E) Relevance network of the multi-omics signatures.

## Discussion

In multi-omics studies, unraveling the mechanisms underlying complex traits, such as fat deposition, is often challenged by highly intertwined genotype-by-environment (G × E) interactions. The hologenomic perspective allows researchers to overcome the limitations of single-dimensional analyses, systematically characterizing holistic level variation and its underlying statistical inferences (Lappalainen et al., 2024). Utilizing a strictly controlled dietary cross-fostering model, this study aimed to elucidate the mechanisms driving phenotypic divergence in fat deposition between JH and LY pigs. Although environmental factors partially reshaped the microbial community during early development (T1), host genetic effects exerted a stronger driving force as development progressed into the critical phase (T2), establishing stable population-level biological trends. While future studies could enhance statistical power by expanding individual sample sizes, the current results represent quintessential hologenomic signatures distinguishing the two breeds.

Oleic acid (OA) is a monounsaturated fatty acid containing 18 carbon atoms and one cis-double bond, present in nearly all vegetable oils and animal fats (Lim et al., 2013). As a dietary nutrient, OA participates in the co-metabolism between the host and the microbiota (Liu et al., 2025). In our study, OA emerged as a core differential metabolite, maintaining high abundance in the gut and tissues of JH pigs. Literature indicates that high oleic acid intake robustly activates adipocyte precursor cells (APCs), driving their rapid proliferation to supply ample substrates for intramuscular and subcutaneous fat deposition (Wing et al., 2025). It also regulates lipid metabolism by activating specific G protein-coupled receptors, such as GPR120 (Du et al., 2022). Furthermore, OA enhances insulin signaling, promoting insulin-induced glucose uptake in adipocytes, and stimulates adipogenesis and the expression of downstream targets via PPARγ activation, ultimately inducing triglyceride accumulation in 3 T3-L1 cells (Xu et al., 2024). Additionally, OA has been shown to alleviate endoplasmic reticulum stress triggered by high-fat intake or massive adipose expansion, functioning as a potent lipogenic molecule (Castillo-Quan et al., 2023; Ali et al., 2025). These biological roles are highly consistent with the robust and healthy adipose expansion phenotype observed in JH pigs.

Our results demonstrated that the genera *Eubacterium* sp. *AM28-29* and *Blautia* were significantly and positively correlated with backfat thickness and oleic acid levels, suggesting their active roles in the production, bioconversion or intestinal metabolism of OA. Previous studies have identified *Eubacterium* and *Blautia* genera in the gut environments of various mammals, primarily implicating them in complex carbohydrate degradation and secondary lipid metabolism (Hosomi et al., 2022; Chanda et al., 2024). Notably, the genus *Blautia* possesses a robust capacity to produce SCFAs, which promote adipocyte differentiation and lipogenesis in preadipocytes through GPR43 (Mann et al., 2024). Integrating these findings with our multi-omics data, we postulate that these genera, specifically enriched in JH pigs, not only possess strong lipid substrate processing capabilities but also function as key synergistic microbes that convert nutrients into lipogenic substrates like OA. Furthermore, the significant negative correlation between these genera and the inhibitory metabolite OEA further indicates their deep involvement in constructing the pro-adipogenic microenvironment in JH pigs.

*In vivo*, oleoylethanolamide (OEA) primarily functions as a lipid messenger that inhibits lipogenesis in adipose tissue and promotes mitochondrial *β*-oxidation of fatty acids by activating the PPARα receptor (Chen et al., 2015; Seguella et al., 2025). Lipid metabolism requires not only substrate synthesis but also transmembrane transport (Li et al., 2026). OEA signaling stimulates this oxidative breakdown process, transmitting satiety signals to the central nervous system and reducing food intake (Brown et al., 2017; Cheng et al., 2024). Consequently, OEA is generally considered to enhance fatty acid utilization and acts as a brake against excessive fat accumulation (Tutunchi et al., 2020). Previous studies have suggested that reduced OEA levels may weaken satiety-related signaling and thereby favor continued energy intake. Although feed intake and feeding behavior were not measured in the present study, this mechanism may provide a possible explanation for the observed association between reduced OEA levels and increased fat deposition.

The *APOD* gene plays a crucial role in lipid transport, metabolism and protection against oxidative stress (Jiang et al., 2025; Desmarais et al., 2018). Structurally, as an atypical lipocalin, *APOD* is a member of the lipocalin superfamily and is closely associated with obesity, dyslipidemia, and systemic metabolic reprogramming (Rassart et al., 2020). Studies have found that enrichment of the *APOD* gene in muscle cells can promote increased intramuscular fat (Sun et al., 2024). Our identification of *APOD* as a candidate gene at the intersection of transcriptomic DEGs and high-*F*_ST_ genomic regions, combined with eQTL evidence linking the 13_133481393_G allele to higher *APOD* transcript levels, provides a multi-level basis for prioritizing this locus in future functional studies. The near-fixation of the G allele in JH pigs and the A allele in LY pigs represents a striking population-level divergence consistent with historical selective breeding for divergent fat deposition capacity. We propose a working hypothesis that high *APOD* expression in JH pigs may contribute to a transcriptomic landscape favoring lipid synthesis, which in turn may be associated with the elevated OA and reduced OEA levels observed in the gut. This hypothesis, while supported by convergent multi-omics associations, is not yet substantiated by direct experimental evidence. Future studies employing *APOD* overexpression or knockout pig models and intestinal organoid systems would be valuable for testing this model.

Ultimately, this study provides correlative insights into the genetic characteristics of Chinese indigenous pig breeds and offers novel targets and strategies for modulating meat quality traits in livestock through precision breeding and nutritional interventions.

### Limitations

A limitation of this study is that muscle eQTL data were used to support the prioritization of host candidate genes in an investigation centered on the intestinal microbiome. Because eQTL effects may vary substantially among tissues, regulatory associations identified in skeletal muscle may not reflect those occurring in cecal or other intestinal tissues. Therefore, the muscle eQTL findings should be regarded as complementary evidence related to systemic metabolism and fat deposition rather than as direct evidence of intestinal gene regulation. Validation using tissue-matched intestinal eQTL data will be necessary in future studies.

## Conclusion

In summary, this study integrates genomic, transcriptomic, metagenomic, and metabolomic data to characterize the hologenomic signatures underlying the robust fat deposition phenotype of Jinhua pigs. Our findings reveal convergent associations across multiple analytical layers pointing toward a candidate biological axis involving *APOD* genetic divergence, an intestinal metabolic microenvironment enriched in OA and depleted of OEA, and the selective enrichment of lipid-associated gut microbiota including *Eubacterium* sp. *AM28-29* and *Blautia*. We propose a working model in which fixation of the 13_133481393_G allele at the *APOD* locus is associated with elevated *APOD* expression and a transcriptomic landscape favoring lipid synthesis, which may in turn shape a gut niche that is permissive for fat accumulation and selectively enriched for synergistic microbiota. These microbial taxa may further reinforce the OA–OEA imbalance, potentially stabilizing a pro-adipogenic intestinal environment. We emphasize that these conclusions are based on multi-omics association analyses and represent hypotheses to be tested by future functional experiments, including *APOD* gain- and loss-of-function animal models and targeted microbial colonization studies. The candidate targets and mechanistic hypotheses generated by this study provide a rational foundation for future research into host–microbe interactions governing complex performance traits, and may inform strategies for precision breeding and microbiota-based modulation of meat quality in livestock.

## Materials and methods

### Animals and experimental design

A total of 60 pigs, including 30 JH pigs and 30 LY pigs, were included in the study. The experiment comprised three growth stages corresponding to 60, 90 and 180 days of age. At each stage, 10 JH pigs and 10 LY pigs were included, resulting in 20 pigs per stage. All animals were raised under standardized environmental and management conditions at the same commercial pig farm in Zhejiang Province, China.

After weaning at 21 days of age, pigs of each breed were randomly allocated to one of two dietary treatments. In the breed-specific diet group, fifteen JH pigs and fifteen LY pigs were fed according to the feeding standards established for their respective breeds. In the reciprocal diet-swap group, an additional fifteen JH pigs were fed the LY diet, whereas fifteen LY pigs were fed the JH diet. All pigs received their assigned experimental diets from weaning until slaughter at the designated age. This reciprocal feeding design allowed comparisons between the two breeds under matched dietary conditions and helped distinguish breed-related effects from dietary effects.

Separate groups of pigs were slaughtered at 60, 90 and 180 days of age. Cecal tissue and cecal content samples were collected immediately after slaughter for subsequent analyses. Carcass weight and three-point backfat thickness were measured. The samples were quickly transferred into 2 mL sterile cryovials and rapidly frozen with liquid nitrogen to preserve the integrity of the samples. They were then stored at −80 °C for future analysis.

### Meat quality assessment

Meat color was evaluated 1 to 2 h postmortem using samples of the longissimus dorsi muscle collected at the thoracolumbar junction. Each sample was divided into two equal portions and placed flat on a white porcelain plate. Visual assessment was conducted under natural light by comparing the samples with standardized reference meat samples and a six-point meat color scale. A score of 1 indicated pale, soft, and exudative meat, with a color ranging from very pale pinkish white to white. A score of 2 indicated mildly pale, soft, and exudative meat with a pale grayish-red color. Scores of 3 and 4 represented normal meat color, corresponding to bright red and dark red, respectively. A score of 5 indicated mildly dark, firm, and dry meat with a light purplish-red color, whereas a score of 6 indicated dark, firm, and dry meat with a dark purplish-red color. Intermediate scores at 0.5-point intervals were allowed between adjacent integer categories, and the final score was recorded.

The meat samples were stored at 4 °C for 24 h and then divided into two portions and placed on a white porcelain plate. Marbling was visually evaluated using a six-point scoring scale. Intermediate scores of 0.5 were assigned when the degree of marbling fell between two adjacent integer categories. A score of 1 indicated almost no visible marbling, whereas a score of 2 indicated a small amount of visible marbling. Scores of 3 and 4 indicated sparse and moderately apparent marbling, respectively. A score of 5 indicated clearly visible marbling, and a score of 6 indicated pronounced and dense marbling.

### Metagenomic sequencing and analysis

The QIAamp Fast DNA Stool Mini Kit (Qiagen, Germany) was applied to extract the microbial DNA from the content samples of cecum. The amount and quality of DNA were measured using a NanoDrop 1,000 spectrophotometer (NanoDrop Technologies, Wilmington, DE, USA), and sterile water was used to adjust the DNA concentration to a final concentration of 1 ng/μL. The library was generated using the Illumina TruSeq™ DNA Sample Preparation Kit, followed by metagenomic shotgun sequencing performed by Novogene (Beijing, China) on the Illumina HiSeq 2,500 platform. Each sample was sequenced to a depth of 10 GB with 150 bp paired-end reads.

Raw sequencing reads were quality controlled using fastp (v0.20.0) (Chen et al., 2018), including adapter trimming and removal of low-quality reads. The quality-filtered reads were subsequently aligned to the pig reference genome *Sus scrofa* 11.1 using Bowtie2 (v2.3.5.1) (Langmead and Salzberg, 2012; Warr et al., 2020). Reads mapped to the host genome were discarded, and the remaining non-host reads were retained for taxonomic profiling. A pre-built Kraken2 database based on the representative genomes from the Genome Taxonomy Database (GTDB) release 207 was used for taxonomic classification (Parks et al., 2022). The non-host reads from each sample were classified against this database using Kraken2 (Lu et al., 2022). Bracken (Lu et al., 2017) was then applied to the Kraken2 classification results to re-estimate microbial abundances at the species level (Supplementary Table 9).

### RNA extraction and quantification

Using the TRizol reagent, the total RNA of the corresponding all tissues was prepared for mRNA sequencing. The RNA integrity and yield were assessed by the RNA Nano 6,000 Assay Kit of the Bioanalyzer 2,100 system (Agilent Technologies, Santa Clara, CA, United States) and the NanoPhotometer spectrophotometer (IMPLEN, Westlake Village, CA, United States). 3 μg of RNA per sample was used to create sequencing libraries using the NEBNext Ultra TM RNA Library Prep Kit for Illumina (NEB, Ipswich, MA, United States) in accordance with the manufacturer’s instructions. Index numbers were added to identify each sample’s sequences. Finally, the clustered libraries were sequenced on an Illumina HiSeq platform in Novogene (Beijing, China), and 150-bp paired-end reads were generated.

Using fastp (v0.20.0) for quality control, reads containing adapters, with an N base proportion greater than 0.002, or low-quality bases exceeding 50% were removed (Chen et al., 2018). HISAT2 (v2.2.1) (Kim et al., 2019) was used to align the clean data to the pig reference genome *Sus scrofa* 11.1 (Warr et al., 2020), retaining samples with a mapping rate greater than 90%. StringTie (v2.1.7) was employed to assemble transcripts and quantify gene expression, resulting in a gene expression matrix after validation with gffcompare (Pertea et al., 2015; Supplementary Table 10). To make the estimated gene expression comparable across genes and different experimental conditions, TPM (transcripts per kilobase million) values were calculated for subsequent data analysis (Supplementary Table 11).

### Metabolome analysis

The LC–MS analysis of the sample was conducted with an Hypesil Gold column (100 × 2.1 mm, 1.9 μm; Thermo Fisher, USA). Under both positive and negative ion modes, Q Exactive™ HF (Thermo Fisher, Germany) and Vanquish UHPLC (Thermo Fisher, Germany) was performed to identify metabolites eluted from the column. Import the raw files (.raw) obtained from mass spectrometry detection into Compound Discoverer 3.1 software for spectrum processing and database searching to obtain the qualitative and quantitative results of metabolites. Metabolites with a coefficient of variance (CV) below 30% in the QC sample were retained as the final identification results for subsequent analysis. Metabolites were annotated using the KEGG database (Ogata et al., 1999), HMDB database (Wishart et al., 2007) and LIPID database (Cotter et al., 2006; Supplementary Table 12).

#### Natural variations and population genetic analysis

Using the plink (v1.90) (Chang et al., 2015) software, genotype data for 289 JH pigs and 795 imported pigs (244 Yorkshire, 509 Landrace, and 41 Landrace×Yorkshire pigs) were obtained from the PHARPV4 database for natural variation analysis (Wang Q. et al., 2025). Based on the parameters --geno 0.9 --maf 0.01, a preliminary filtering of the genomic data is performed. VCF format files for population selection analysis were processed using VCFtools (0.1.16) (Danecek et al., 2011) to calculate Fixation Index (*F*_ST_), using a 40-kb window size with a 20-kb window-step size across the genome (Lu et al., 2013). Finally, the top 1% of genes were selected for annotation for the subsequent analysis.

### Statistical analysis

#### Phenotypic data analysis

Phenotypic traits, including BFT, carcass weight and marbling scores, were analyzed using R (v4.4.3). To assess the independent and interactive effects of breed, diet, and age on fat deposition, a three-way ANOVA was performed. Prior to three-way ANOVA, the normality of model residuals was verified using the Shapiro–Wilk test for both backfat thickness (W = 0.964, *p* = 0.235) and carcass weight (W = 0.969, *p* = 0.337), confirming that the parametric assumptions were met. For pairwise comparisons between breeds (JH and LY) under specific time points or dietary treatments, statistical significance was determined using the Wilcoxon rank-sum test. A *p*-value < 0.05 was considered statistically significant.

#### Transcriptomic data processing and co-expression network analysis

DESeq2 (v1.42.0) was employed both for differential gene expression analysis, based on a negative binomial generalized linear model, and for PCA to visualize global expression patterns. The significance thresholds for DEGs were set at an adjusted *p*-value < 0.05 and |log₂ FC| ≥ 1.

Additionally, WGCNA was performed using the WGCNA R package to identify gene modules highly correlated with breed and BFT traits (Langfelder and Horvath, 2008). Gene expression levels (TPM) were used as input for network construction. To ensure robust analysis, genes with low expression levels were filtered out; specifically, only genes expressed (TPM > 0.5) in at least 20% of the total samples were retained. The expression data were then log_2_(TPM + 1) transformed. To focus on the most biologically informative variation, the top 5,000 genes ranked by Median Absolute Deviation (MAD) were selected for subsequent analysis. The goodSamplesGenes function in the WGCNA R package was utilized to check for missing data and ensure data integrity. Hierarchical clustering based on the Euclidean distance of samples was performed to visualize sample relationships. Based on the clustering dendrogram and phenotypic metadata, outlier samples were identified and removed to minimize experimental noise and improve the stability of the co-expression network.

Gene Ontology (GO) and KEGG pathway enrichment analyses for module genes and DEGs were executed using the clusterProfiler and enrichplot packages.

#### Identification and functional analysis of differential metabolites

For metabolomics data at the T2 stage, the OPLS-DA was conducted to evaluate metabolic profiling differences. Data were log-transformed and Pareto-scaled (Wang et al., 2023), followed by differential metabolite analysis using MetaboAnalyst 6.0 (Pang et al., 2024). Differential metabolites between JH and LY pigs under the two dietary conditions were identified based on Variable Importance in Projection (VIP) > 1 and a t-test *p*-value < 0.05. To eliminate diet-induced environmental noise, an intersection strategy was employed for robust, diet-independent, breed-characteristic metabolites. Metabolites showing inconsistent expression patterns across diets were manually excluded. Pearson correlation coefficients between robust metabolites and phenotypic traits (BFT, carcass weight) were calculated and visualized using the pheatmap packages.

#### Microbiome diversity and multivariable association analysis

Beta-diversity of the gut microbiota was evaluated using PCoA based on Bray-Curtis distances. The variance explained by host genetics and diet was quantified using Three-way ANOVA via the vegan R package. To identify specific microbial taxa driving breed differences, the MaAsLin2 package was applied (Mallick et al., 2021). Microbes with low relative abundance (< 0.0001%) and low prevalence (present in < 50% of samples) were filtered out prior to analysis. Taxa with an FDR < 0.05 were considered significantly associated with the breed. Pearson correlation coefficients between species and metabolites were calculated using the cor.test function in R.

#### Mediation analysis

To dissect the causal pathways linking gut microbiota, host metabolism, and phenotypic outcomes, we performed mediation analysis using the mediation package (v4.5.0) in R (Imai et al., 2010). For validation of specific hypotheses, linear models were constructed to assess: (i) the effect of microbial abundance on metabolite levels (mediator model), and (ii) the joint effects of microbial abundance and metabolites on the phenotype (outcome model). Significance of indirect effects was determined by bootstrap inference with 1,000 simulations. Pathways with significant mediation (ACME *p* < 0.05) were retained and ranked by the proportion of effect mediated.

#### Multi-omics integration via DIABLO

To integrate the colonic transcriptomics, metabolomics, and microbiome data, we employed the DIABLO framework implemented in the mixOmics R package (Rohart et al., 2017). Prior to integration, each omic dataset underwent specific normalization. The transcriptomic data (TPM values of genes intersecting the WGCNA greenyellow module and *F*st) were log2(TPM + 1) transformed. To account for the compositional nature of the metagenomic data, microbial abundances were subjected to CLR transformation using the compositions package. To ensure data consistency, only common samples across all four datasets (mRNA, metabolites, microbes and breed factors) were retained for the downstream analysis. A supervised multi-block partial least squares discriminant analysis (N-integration) was performed using the block.splsda function. The breed (JH and LY) was utilized as the categorical outcome variable (Y). A design matrix was established with off-diagonal elements set to 0.1, prioritizing the discrimination between breeds while maintaining the correlation between omics layers. The final model was constructed with two latent components per block. The cross-omic correlations were visualized using circos plots and relevance networks. A stringent similarity threshold (cutoff = 0.7) was applied to filter the strongest associations between genes, metabolites and microbial taxa. The relevance network was generated based on the similarity matrix derived from the DIABLO model, with node colors representing different omics layers and edge colors indicating the direction of correlation.

## Data Availability

The metagenomic data presented in the study are deposited in the NCBI repository, accession number PRJNA1518402.
